# Assessment of the occurrence, spatiotemporal variations and geoaccumulation of fifty-two inorganic elements in sewage sludge: A sludge management revisit

**DOI:** 10.1038/s41598-017-05879-9

**Published:** 2017-07-18

**Authors:** Fidèle Suanon, Qian Sun, Xiaoyong Yang, Qiaoqiao Chi, Sikandar I. Mulla, Daouda Mama, Chang-Ping Yu

**Affiliations:** 1 CAS Key Laboratory of Urban Pollutant Conversion, Institute of Urban Environment, Chinese Academy of Sciences, Xiamen, 361021 China; 2 University of Chinese Academy of Sciences, Beijing, 100049 China; 3 Laboratory of Physical Chemistry, University of Abomey-Calavi, 01 BP: 4521 Cotonou, Benin; 4 Laboratory of Inorganic Chemistry and Environment, University of Abomey-Calavi, 01 BP: 4521 Cotonou, Benin; 5 Graduate Institute of Environmental Engineering, National Taiwan University, Taipei, 106 Taiwan

## Abstract

The limited information about the sludge quality has made its management a top environmental challenge. In the present study, occurrence and the spatiotemporal variations of 52 inorganic elements were investigated in the sludge samples from three wastewater treatment plants (WWTPs) in Xiamen city, China. The results showed, the occurrence of 49 elements with the concentrations in the range of >125–53500 mg kg^−1^ dry sludge (DS) for commonly used industrial metals, 1.22–14.0 mg kg^−1^ DS for precious metals, and 1.12–439.0 mg kg^−1^ DS for rare earth elements. The geo-accumulation studies indicated a moderate to high levels of buildup of some elements in the sewage sludge. Principal components analysis (PCA) indicated strong spatial and weak temporal variations in the concentrations of the elements. Therefore, the sludge disposal operations, based on the element concentrations, geoaccumulation and economic potential are suggested for each WWTP. Sludge from W1 and W2 were found suitable for agricultural usage, while that from W3 showed a higher economic potential for the recovery of precious metals. This study concludes that a comprehensive analysis of the elements in the sewage sludge could provide critical information for the disposal and management of the sludge.

## Introduction

China is a populous country with a population of over 1.3 billion. With the rapid industrialization, urbanization, and economic growth, China is facing growing environmental issues^1–4^, including the production and disposal of huge amounts of sewage sludge. According to NBSC^5^ and Yang *et al*.^6^, 6.25 million tons dry sludge (DS) was produced in 2013 in China, of which, >84% was disposed improperly. The safe disposal of the municipal sewage sludge has become a major environmental challenge in China and in many countries of the world^7–9^.

Sewage sludge is a rich source of phosphorous and nitrogen, and could be value-added as fertilizer^10, 11^. However, the high concentration of metals in sludge is a great concern^12–14^, that may pose adverse impact on human and other living organisms when their bioavailability exceeds the threshold limit (for example, 456, 43, 1099, 263 and 133 mg kg^−1^ for Cu, Ni, Zn, Cr, and Pb, respectively)^15, 16^. These metals mainly originate from the aqueous phase of the wastewater, and then concentrate in the sludge during the treatment processes like precipitation, coagulation, adsorption etc. For the sludge with higher levels of metals, the resource recovery, including the major industrial elements, precious metals, and rare earth elements, has been suggested as an alternative, not only to reduce the pollutant loadings but also to exploit the economic potential of the sludge before its disposal^17, 18^. The sewage sludge, which is the end-of-life product, would now be considered the “urban mines”. Therefore, it is necessary to investigate the loads of the metallic elements to achieve the proper disposal of sewage sludge^7–9^.

Several studies have reported the occurrence of metallic elements, mainly heavy metals in the sewage sludge in China. Yang *et al*.^19^ collected sludge samples from 107 municipal wastewater treatment plants (WWTPs) located in 48 cities in China in 2006. The results showed that the average concentrations of As, Cd, Cr, Cu, Hg, Ni, Pb, and Zn were 20.2, 1.97, 93.1, 218.8, 2.13, 48.7, 72.3, and 1058 mg·kg^−1^, respectively. Dong *et al*.^20^ reported the heavy metal occurrence in sewage sludge in China, with concentrations up to 293.7, 181.7, 114.8, 40.3, 1453.9 mg kg^−1^ DS for Cr, Cu, Ni, Pb and Zn, respectively. In another study, carried out on industrial sewage sludge, Wu *et al*.^21^ reported the concentrations up to 172300, 237, 2225, and 1700 mg kg^−1^ DS for Cd, Cu Ni and Zn, respectively. In addition, Suanon *et al*.^22, 23^ reported concentration of 64, 73.1, 604.1, 1102.1, 483.9, and 2060.3 mg kg^−1^ DS for Cd, Co, Cu, Cr, Ni and Zn, respectively, in the urban sewage sludge. As it can be noticed that most of the studies investigated a limited number of metals, which are generally with greater environmental concerns, and they are frequently listed in the environmental monitoring methods, standards, or regulations^24, 25^. In order to better assess the environmental and ecotoxicological risks and to appreciate the economic opportinuity for their recovery, it is necessary to investigate a broad range of elements in the sludge.

Therefore, the present work aimed to (1) investigate the occurrence of 52 elements, including industrial metals, precious metals, rare earth elements and phosphorous in sewage sludge, (2) understand the temporal and spatial variations, (3) appreciate the quality of sludge and evaluate the geoaccumulation index, and (4) to indicate the proper sludge disposal methods. To our knowledge, this is the first investigation on such a broad range of inorganic elements in the sewage sludge in China.

## Materials and Methods

### Study area

Three municipal WWTPs (W1-3) located in Xiamen, China were investigated in this study. The wastewater in W1 was nearly 100% domestic wastewater, while in W2 and W3, the industrial wastewater contributed to 20% and 50%, respectively along with domestic wastewater. W1, W2, and W3 served around 1.0, 0.35, and 0.30 million inhabitants, respectively^26^. The average daily excess sludge was 1691.7, 214.5, and 243.7 tones, with the dry weight of 53.3, 4.5 and 8.1 tones, respectively for W1, W2 and W3^26^. The treatment process varied from one plant to another. Briefly, in W1, the treatment included screening, grit chamber, biological aerated filter, and UV disinfection. In W2, screening, grit chamber, orbal oxidation ditch, secondary settling tank, and UV disinfection were involved; while in W3, screening, grit chamber hydrolyzing pond, anaerobic/anoxic/oxic (A2O) reactor, secondary settling tank, and chemical disinfection were the main treatment processes^26^.

### Sludge sampling and pretreatment

Activated sludge samples in W1 and the return sludge samples in W2 and W3 were seasonally collected from each WWTP on February 20^th^ (F), May 8^th^ (M), August 11^th^ (A), and November 12^th^ (N) 2014^26^. Sludge samples were concentrated by centrifugation (5000 g) at 4 °C for 15 min, and dried via lyophilization. The dried sludge was then grounded in clean mortar and sieved by passing through a mesh size <0.15 mm. The obtained powders were kept in polyethylene bags for physicochemical characterization and determination of elements.

### Physicochemical characterization

Physicochemical parameters, including pH, electrical conductivity (EC), and C, N, and S, were analysis. The values of pH and EC were determined in the filtrate of dissolved sludge sample in the milliQ water at ratio 1/5 (v/v)^27^ using a multi-parameter meter (HACH, HQ40d). The total carbon (TC), total nitrogen (TN), and total sulfur (TS) were determined by a macro elemental CNHS/O Analyzer (Vario MAX; Elementar, Germany). The pre-detection of the elements in the sludge samples was performed using scanning electron microscopy (SEM, HITACHI S-4800) with energy dispersive X-ray (EDX) spectroscopy (Genesis XM2).

### Metals contents in the sludge

#### Vessels and cleanup procedure

All digestion vessels (polytetrafluoroethylene) were cleaned by leaching with hot 10 mL aqua regia (mixture of HNO_3_ and HCl at the ratio of 3:1) at 90 °C for a minimum of 2 h according to the European Norm 3051A^28^. The cleanup was then completed following the recommendations by Westerhoff *et al*.^17^. All volumetric wares, including glass calibrate cylinders, were carefully acid washed and rinsed following the same recommendations. The nitric acid and hydrochloride acid were analytical grade (Merck KGaA, Darmstardt Germany).

#### Sludge digestion and quality control (QC)

Sludge sample (0.100 g) and freshly prepared aqua regia (12.0 mL) were placed in the digestion tube. The reaction mixture was kept for 30 min at room temperature, and then the digestion was completed under microwave at 180 °C according to the European Norm 3051A^28^. The digestion program is described in the Table S1 in the SI. After cooling, the samples were filtrated using a Millipore filter (0.45 µm) and collected in a 50 mL polypropylene centrifugation tube and diluted to 50.0 mL with 2% nitric acid solution. Samples were kept at 4 °C prior to the analysis. For quality control, an instrument blank, procedural blank and certified reference material samples with known concentrations of elements (GBW07309, GSD-9, Inspection and Quarantine of the People’s Republic of China) were applied for each sample batch. All experiments were performed in triplicate.

#### Metal Detection

Inductively coupled plasma mass spectroscopy (ICP-MS, Agilent 7500CX) and inductively coupled plasma optical emission spectrometry (ICP-OES, PerkinElmer Optima 7000 DV, USA) were used for the elemental quantification. Stock solutions of element standards were purchased from National Center of Analysis and Testing for Nonferrous Metals and Electronic Materials (NCATN). The target elements were analyzed in four groups: G1 [(GNM-M27867-2013) including Ag, Al, As, Ba, Ca, Cd, Co, Cr, Cs, Cu, Fe, Ga, K, Mg, Mn, Mo, Na, Ni, P, Pb, Rb, Re, Sb, Sr, Tl, V and Zn], G2 [(GNM-M16181-2013) including La, Ce, Pr, Nd, Sm, Eu, Gd, Tb, Dy, Ho, Er, Tm, Yb, Lu, Y and Sc], G3 [(GSB 04-1769-2004) including Au, Pd, Pt, Ir and Ru], and G4 [(GNM-M05868-2013) including Hf, Nb, Sn, Ti and W]. The detection limits and the recoveries of target elements are shown in Table S2.

### Statistical analysis

Principal components analysis, conducted by R software (version 3.2.2), was used to identify spatial and temporal variability of the 52 elements in the sewage sludge. Data were first normalized using the vegan package to alleviate negative effect caused by some elements which distributed extremely non-uniform. The correlation among multiple elements was carried out using Pearson’s correlation coefficient assay in SPSS 21.0. Results obtained were then used to construct a heatmap using R software with heatmap package.

## Results and Discussions

### Physicochemical characterization of the sludge

**Figure 1:**
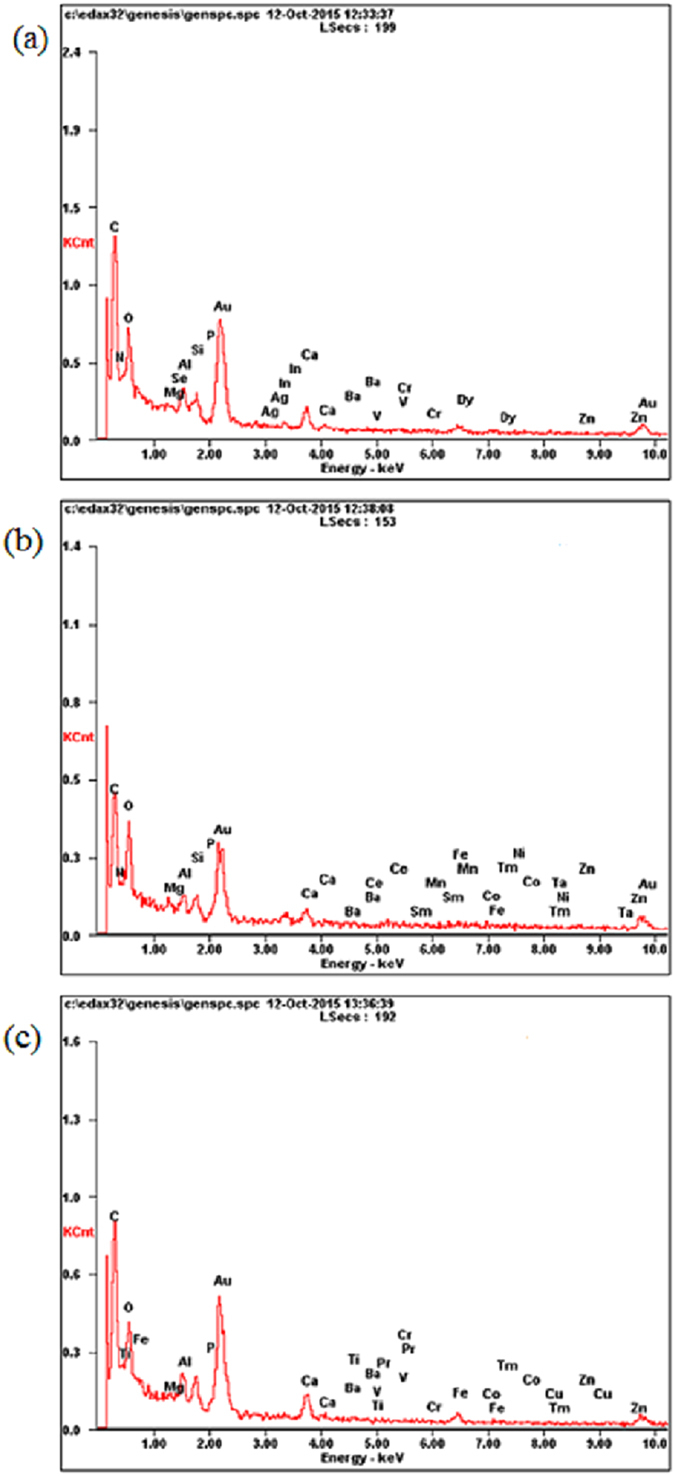
EDX images of sludge samples from W1 (**a**), W2 (**b**) and W3 (**c**) collected in February 2014.

The physicochemical parameters of sludge are shown in Table 1. The pH values of the sludge samples were all close to the neutrality and did not show any significant variations during the four sampling seasons (*p* > 0.05). However, the pH values in W3 were higher than W1 (*p = *0.014). The relatively high values of EC (0.73–1.81 mS cm^−1^) indicated the high concentration of charged particles such as Cl^−^, Na^+^, K^+^, NH_4_^+^, NO_3_^−^, SO_4_^2−^, HCO_3_^−^ etc. in the sludge samples^29^. Sludge samples were C, N and S-rich, with the average concentrations of 260.4, 36.5, and 22.3 g kg^−1^ DS, respectively. The EDX image (Fig. 1) emphasizes the presence of a broad range of elements, including Fe, Al, Mg, Ca, Mn, Zn, P, V, Ba, Cr, Zn, Ag, etc. in the sludge samples. To be noticed, the high peak of gold observed in Fig. 1 is due to the coating process for the SEM/EDX analysis.

**Table 1:** Physicochemical characterization of wastewater and sludge samples during the four seasons.

WWTPs	pH	EC (mS cm^−1^)	C (g kg^−1^)	N (g kg^−1^)	S (g kg^−1^)
W1-F	6.8	1.23	205.5	25.12	32.37
W1-M	6.8	1.71	346.0	40.86	16.36
W1-A	6.9	1.74	313.0	30.86	14.36
W1-N	6.8	1.81	306.8	43.86	13.36
W2-F	6.7	1.53	270.9	47.36	14.11
W2-M	6.9	1.02	246.1	38.87	18.89
W2-A	7.1	1.04	316.0	36.91	37.70
W2-N	6.9	1.27	241.0	40.66	16.35
W3-F	6.9	0.73	257.3	42.41	28.04
W3-M	7.1	1.15	237.2	27.86	29.03
W3-A	7.2	0.95	125.9	21.59	14.54
W3-N	7.2	0.92	259.6	41.83	32.55

### Concentration of target elements in the sludge

#### Major industrial elements

Out of 52 investigated elements, 49 were detected in the sludge. The most widely used industrial elements like Al, Fe, P, Ca, K, Mg, Na, Mn and W etc. were detected at very high concentrations in the sludge samples with an average annual concentration ranging from 125 mg kg^−1^ DS (W) to 53500 mg kg^−1^ DS (Fe) (Table 2). Concentrations of Ti, Ba, Sr, Zn, Cu, Sn, Ni, and Cr were relatively lower compared to the above-mentioned elements in the sludge, with concentrations ranged from 14.9 mg kg^−1^ DS (for Ni, in W1) to 2230 mg kg^−1^ DS (for Zn in W3). Regarding the elements with great environmental concerns such as As, Cd, Co, Cr, Cu, Ni, Pb, and Zn, the concentrations ranged from 3.9–5.2 mg kg^−1^ DS, 9.1–61.4 mg kg^−1^ DS, 5.46–48.7 mg kg^−1^ DS, 36.2–1600.0 mg kg^−1^ DS, 64.8–1390.0 mg kg^−1^ DS, 14.9–610.0 mg kg^−1^ DS, 42.8–63.4 mg kg^−1^ DS, and 231–2230 mg kg^−1^ DS, respectively. The concentrations of the major industrial elements were within the similar ranges described in the studies of the domestic sewage sludge in the US^17^ and China^20, 22^.

**Table 2:** Average annual concentrations (mg kg^−1^ DS) of detected elements in the sewage sludge (n = 4).

Elements	W1	W2	W3
**Industrial elements**			
Al	3.54E + 04 ± 3.71E + 03	4.48E + 04 ± 3.57E + 03	3.90E + 04 ± 2.43E + 03
Fe	5.35E + 04 ± 5.06E + 03	3.99E + 04 ± 4.65E + 03	5.04E + 04 ± 9.43E + 02
P	1.47E + 04 ± 5.96E + 02	1.69E + 04 ± 2.76E + 03	3.65E + 04 ± 1.14E + 03
Ca	1.93E + 04 ± 8.14E + 02	1.18E + 04 ± 1.81E + 03	2.00E + 04 ± 8.44E + 02
K	3.53E + 03 ± 3.97E + 02	4.56E + 03 ± 4.72E + 02	2.65E + 03 ± 2.12E + 02
Mg	5.31E + 03 ± 4.50E + 02	3.85E + 03 ± 3.28E + 02	3.96E + 03 ± 5.90E + 02
Na	2.11E + 03 ± 2.68E + 02	2.06E + 03 ± 3.41E + 02	2.87E + 03 ± 2.94E + 02
Mn	6.28E + 02 ± 9.24E + 01	4.20E + 02 ± 5.68E + 01	3.03E + 02 ± 1.90E + 01
W	1.25E + 02 ± 9.40E + 00	3.64E + 03 ± 1.08E + 03	1.16E + 03 ± 3.77E + 02
Ti	7.77E + 02 ± 8.26E + 01	3.71E + 02 ± 2.26E + 01	5.47E + 02 ± 1.05E + 01
Ba	3.79E + 02 ± 1.19E + 01	4.70E + 02 ± 3.50E + 01	9.49E + 02 ± 4.86E + 01
Sr	1.82E + 02 ± 9.12E + 00	1.48E + 02 ± 7.12E + 00	2.10E + 02 ± 3.15E + 00
Zn	4.65E + 02 ± 2.56E + 02	2.31E + 02 ± 3.07E + 01	2.23E + 03 ± 3.54E + 02
Cu	6.48E + 01 ± 6.74E + 00	1.61E + 02 ± 3.21E + 01	1.39E + 03 ± 1.53E + 02
Sn	1.42E + 02 ± 8.44E + 00	2.16E + 02 ± 1.45E + 01	3.98E + 02 ± 3.88E + 01
Ni	1.49E + 01 ± 9.93E-01	5.42E + 01 ± 1.14E + 01	6.10E + 02 ± 6.19E + 01
Cr	3.62E + 01 ± 8.95E-01	9.50E + 01 ± 2.04E + 01	1.60E + 03 ± 2.39E + 02
Ga	3.36E + 01 ± 1.31E + 00	5.75E + 01 ± 2.87E + 00	8.00E + 01 ± 5.87E + 00
Pb	4.28E + 01 ± 1.44E + 00	4.90E + 01 ± 2.49E + 00	6.34E + 01 ± 6.56E + 00
V	1.54E + 01 ± 6.31E-01	1.42E + 01 ± 9.08E-01	1.46E + 01 ± 6.14E-01
Co	5.46E + 00 ± 2.14E + 00	4.87E + 01 ± 1.13E + 01	3.49E + 01 ± 1.72E + 00
As	4.49E + 00 ± 3.18E-01	3.96E + 00 ± 3.81E-01	5.20E + 00 ± 1.42E-01
Rb	3.28E + 01 ± 4.46E + 00	5.18E + 01 ± 9.73E + 00	1.27E + 01 ± 2.96E + 00
Nb	8.48E + 00 ± 1.29E + 00	4.57E + 00 ± 1.32E + 00	4.68E + 00 ± 8.50E-01
Mo	1.70E + 01 ± 4.08E + 00	4.10E + 01 ± 6.46E + 00	3.62E + 01 ± 2.78E + 00
Cd	9.09E + 00 ± 8.11E-01	6.14E + 01 ± 3.03E + 00	5.92E + 01 ± 2.97E + 00
Sb	9.79E-01 ± 9.66E-02	1.09E + 00 ± 3.68E-01	1.86E + 01 ± 1.34E + 00
Hf	2.71E + 00 ± 9.37E-02	2.74E + 00 ± 1.37E-01	4.82E + 00 ± 1.45E + 00
Re	BDL	BDL	BDL
Tl	3.27E-01 ± 1.32E-02	5.41E-01 ± 3.74E-02	3.55E-01 ± 3.03E-02
**Precious elements**			
Pd	2.37E + 00 ± 6.71E-01	2.18E + 00 ± 1.26E-01	1.08E + 01 ± 4.39E-01
Ag	6.08E + 00 ± 2.43E + 00	2.15E + 00 ± 1.64E-01	1.41E + 01 ± 1.83E + 00
Au	1.22E + 00 ± 1.90E-01	1.35E + 00 ± 1.75E-01	5.59E + 00 ± 3.56E-01
Ru	7.11E-02 ± 1.24E-02	4.83E-02 ± 2.57E-03	1.10E-01 ± 2.06E-02
Ir	BDL	BDL	BDL
Pt	2.60E-02 ± 1.62E-03	2.26E-02 ± 3.11E-03	2.17E-02 ± 2.95E-03
**Rare earth elements**			
Ce	7.42E + 01 ± 7.73E + 00	4.39E + 02 ± 1.23E + 02	2.36E + 02 ± 5.13E + 01
Nd	3.81E + 01 ± 3.67E + 00	4.92E + 01 ± 3.09E + 00	7.15E + 01 ± 7.47E + 00
La	3.81E + 01 ± 3.35E + 00	2.55E + 02 ± 4.77E + 01	1.57E + 02 ± 3.37E + 01
Y	2.43E + 01 ± 1.61E + 00	3.26E + 01 ± 2.19E + 00	1.34E + 02 ± 1.17E + 01
Pr	1.04E + 01 ± 9.19E-01	1.34E + 01 ± 8.88E-01	2.22E + 01 ± 4.37E + 00
Sc	2.08E + 00 ± 3.60E-01	3.87E + 00 ± 9.64E-01	0.00E + 00 ± 0.00E + 00
Sm	9.33E + 00 ± 9.81E-01	1.12E + 01 ± 6.72E-01	1.80E + 01 ± 8.94E-01
Gd	1.06E + 01 ± 1.82E + 00	1.46E + 01 ± 1.82E + 00	1.21E + 01 ± 6.02E-01
Dy	6.37E + 00 ± 4.37E-01	7.48E + 00 ± 4.51E-01	8.26E + 00 ± 4.09E-01
Er	3.17E + 00 ± 2.09E-01	4.03E + 00 ± 2.77E-01	4.09E + 00 ± 1.93E-01
Yb	2.98E + 00 ± 2.20E-01	3.86E + 00 ± 2.81E-01	3.86E + 00 ± 2.07E-01
Eu	1.88E + 00 ± 1.78E-01	2.29E + 00 ± 1.31E-01	1.43E + 01 ± 2.26E + 00
Ho	1.12E + 00 ± 8.02E-02	1.39E + 00 ± 8.87E-02	1.39E + 00 ± 6.89E-02
Tb	1.26E + 01 ± 1.31E + 00	1.20E + 01 ± 4.88E-01	1.37E + 01 ± 4.29E-01
Tm	4.43E-01 ± 3.10E-02	5.75E-01 ± 4.18E-02	5.77E-01 ± 3.01E-02
Lu	BDL	BDL	BDL

BDL = below detection limit.

#### Precious elements

Precious metals i.e. Pd, Ag, Au, Ru, and Pt were detected in the sludge samples (Table 2), while the concentration of Re and Ir were below the detection limits (Table S2). Though the precious metal concentrations were lower in the sludge samples compared to the major industrial elements with the concentration ranges between 2.18–10.8 mg kg^−1^ DS, 2.18–14.1 mg kg^−1^ DS, and 1.22–5.59 mg kg^−1^ DS for Pd, Ag and Au, respectively. However, the detected concentrations have economic attraction, considering the critical and precious nature of these metals. The detected Au and Ag concentrations were within the similar concentration range with the samples from US^17^ and Switzerland^18^.

#### Rare earth elements

A total of 16 rare earth elements, including Ce, Nd, La, Y, Pr, Sc, Sm, Gd, Dy, Er, Yb, Eu, Ho, Tb, Tm, and Lu, were also analyzed in the present study. Fifteen were detected, while Lu was below the detection limit (Table S2). As shown in Table 2, the concentrations of rare earth elements ranged from 1.12 mg kg^−1^ DS (Ho) to 439 mg kg^−1^ DS (Ce). The application of rare earth elements in the manufacturing industry was probably the major source to the sewage sludge via wastewater treatment process. As shown in the Yearbook of Xiamen Special Economic Zone^30^, the mechanical and electronic are the most important industries in Xiamen, where the rare earth elements were widely used besides other wide range of technological applications^31^. In addition, it is known that the Chinese rare earth elements reserve represents 50% of the world reserves^32^, which makes China the leader of the world in the rare earth element production^33^. As a consequence, the residues of these metals could accumulate in the sewage sludge.

### Spatiotemporal variation

#### Temporal variation

**Figure 2:**
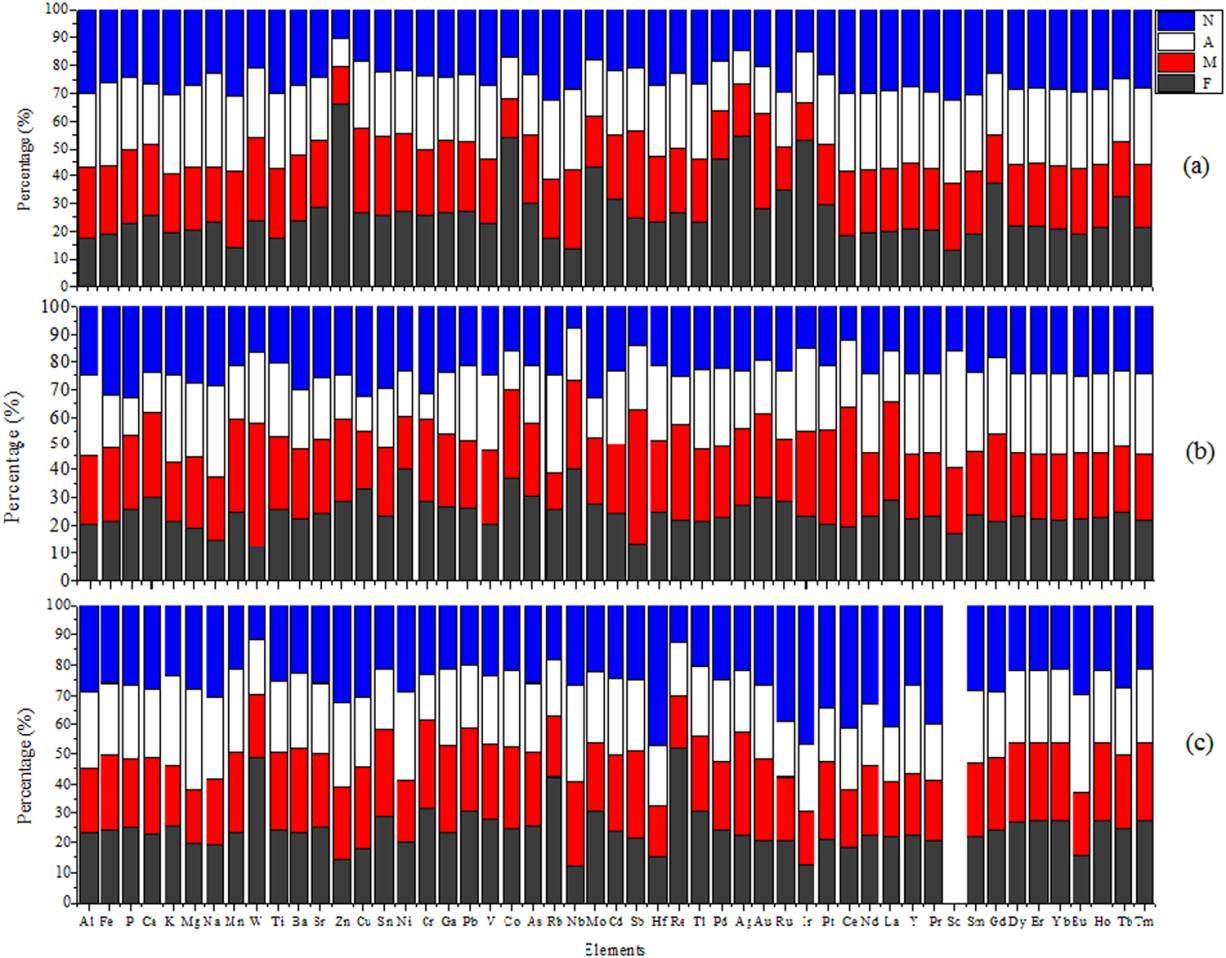
Temporal variations of element concentrations in (**a**) W1, (**b**) W2, and (**c**) W3.

The relative distribution of element concentrations amongst the four seasons, which highlight the temporal variation of the elements, is displayed in Fig. 2. Generally, the concentrations of the most elements within each WWTP did not show significant temporal variation as the elements were nearly equally distributed amongst the four seasons. Particularly, in W3 (Fig. 2c), the equal distribution was more pronounced. However, there were some exceptions. For example, in W1 (Fig. 2a), Mn, W, Zn, Cr, Sb, Re, Pt, Ce, La and Gd exhibited highest concentration in May. In W2 (Fig. 2b), elements generally exhibited lower concentration in August compared to the others seasons.

#### Spatial variation

**Figure 3:**
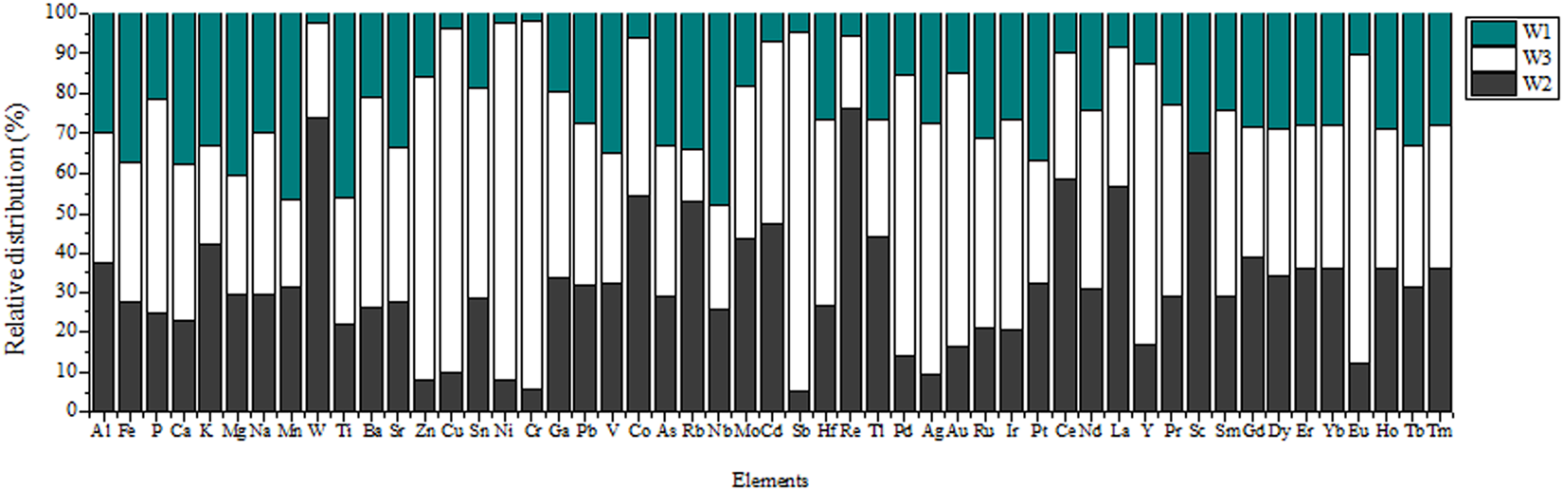
Spatial variations of relative distribution percentages of the target elements.

The relative distribution of each element in W1-3 based on the average concentration from four sampling seasons is shown in Fig. 3 to indicate the spatial variations. Generally, the concentrations of most elements were higher in the sludge samples from W3, followed by W2, while lowest in W1. One-way ANOVA test showed significant spatial variations with *p* < 0.01 for the majority of the elements with the exception of Pr, Dy, Er, Yb, Eu, Tb, and Tm with 0.01 < *p* < 0.05; and Al, Fe, V, Ir, and Pt with *p* > 0.01. The significant spatial variation of the elements was likely due to the difference of the wastewater sources and the difference in the treatment processes in WWTPs. Indeed, the land usage type in W1 is mainly residential or commercial and service, while there are some manufacturing and industrial areas in W2 and W3^26^. The wastewater was nearly 100% domestic wastewater in W1, while W2 and W3 contained 20% and >50% industrial wastewater besides domestic wastewater, respectively. Consequently, the sludge from W3 showed higher concentration of most of the metals, as they are mainly used in the manufacturing and industrial processes^17, 20–22, 34^. In addition, as previously stated, the treatment processes in the WWTPs also vary from one WWTP to another. Biological aerated filters + UV disinfection (BAF + UVdis) were used in W1, Orbal oxidation ditches + UV disinfection (O-OD + UVdis) in W2 while anaerobic/anoxic/oxic + disinfection (A^2^O + dis) were applied in W3 WWTP.

Generally, the concentrations of the elements of greater environmental concern such as Cd, As, Ni, Cr, Cu and Zn in W1 and W2 met with Chinese standard (GB 4284-84, 1983)^25^. The regulated heavy metal concentrations for Cd, Cr, Cu Ni and Zn in the sludge adaptable for agricultural purposes are 5, 600, 800, 100 and 2000 mg kg^−1^ for acidic soils (pH ≤ 6.5) and 20, 1000, 1500, 200 and 3000 mg kg^−1^ for alkaline soil (pH ≥ 6.5), respectively. Therefore, the sludge from W1 and W2 could be used for the land application after properly stabilization. However, the heavy metals accumulation in the soil resulting from the repeated land application, together with the elemental bioavailability and bio-toxicity, needs to be furthered evaluated. In addition, the issue of emerging contaminants and persistent organic pollutants should also be taken into account and investigated during sludge stabilization before any application to the soil. Due to higher heavy metal concentrations, the sludge from W3 cannot be directly applied to the land. Due to the higher concentrations of precious elements in W3, the valuable elements are worthy to be recovered. Therefore, finding cost effective technology for the recovery of precious elements in W3 would be a good alternative.

#### Principal components analysis (PCA)

**Figure 4:**
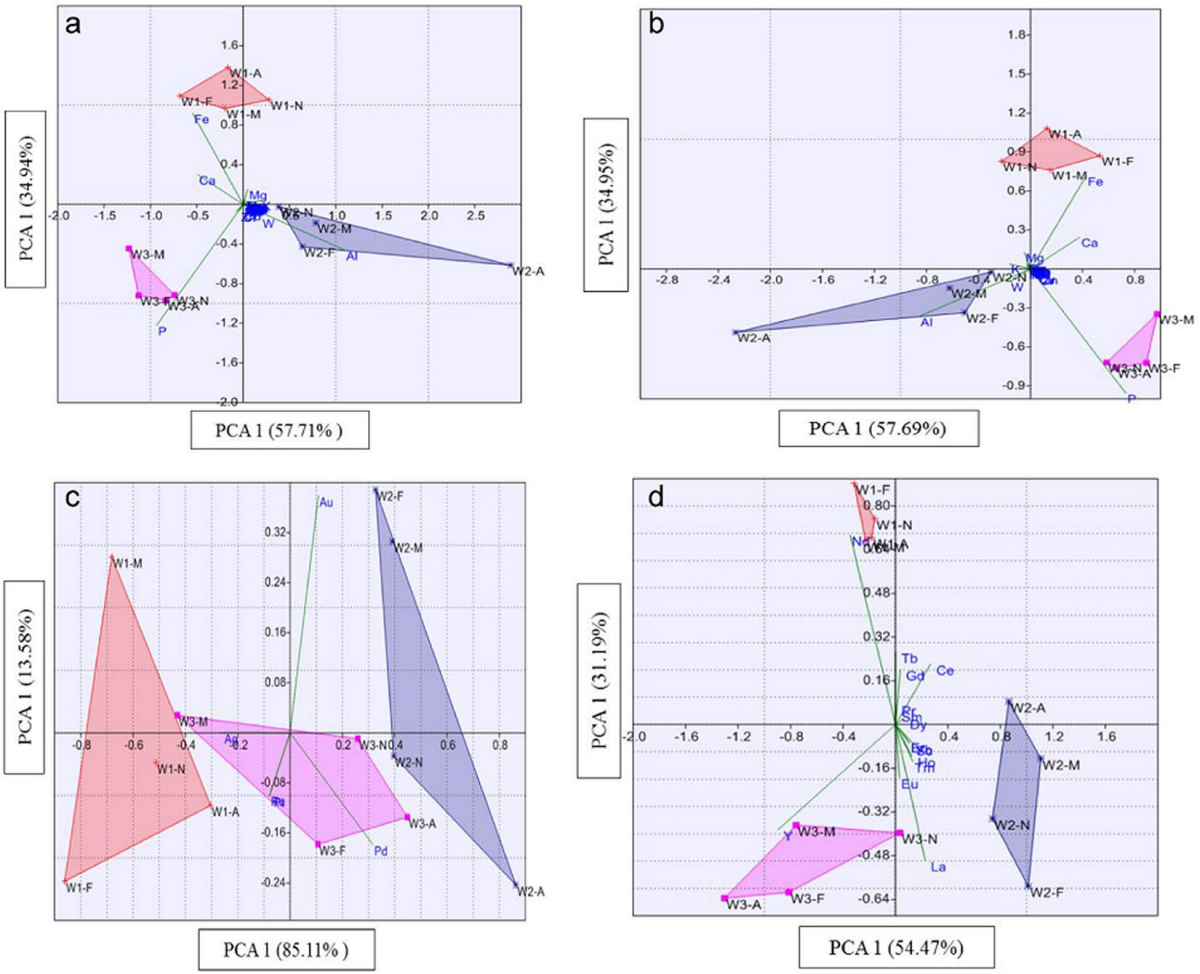
Principal components analysis of (**a**) all detected elements, (**b**) the major industrial elements, (**c**) precious elements and (**d**) rare earth elements.

PCA was performed to show the variation and correlation of the occurrence of elements in different samples. Figure 4a is plotted using the standardized concentrations of all the detected elements, with the principal component 1 (PC1) and PC2 explaining 57.71% and 34.94% of the variance in the elements distribution, respectively. Samples from the same WWTP could be clearly clustered into one group despite the different sampling seasons, indicating the strong spatial variations and little seasonal variations. Most of the elements were around the origin of the coordinates suggesting their minor correlation with PC1 and PC2. However, Al, Ca, P, Fe were far from the origin suggesting that these four elements contributed mostly for PC1 and PC2, which was mainly due to their significantly higher levels (>10^4^ mg kg^−1^), although, the concentrations were standardized. Therefore, PCA was then performed based on the major industrial elements, precious metals, and rare earth elements.

As shown in Fig. 4b,c and d, samples from each WWTP could be clustered into separate groups based on the major industrial elements and rare earth elements. Al, Ca, Fe, and P mainly contributed to the distribution for the major industrial elements, while La, Nd, Ym, and Ce mainly contributed for the variations between samples from W1 and W3 in the case of rare earth elements. The results from PCA, based on the major industrial elements, precious elements and rare earth elements confirmed the high spatial variations and lower temporal variation, which was in accordance with the results in the previous sections.

#### Correlation between target elements and environmental factors

**Figure 5:**
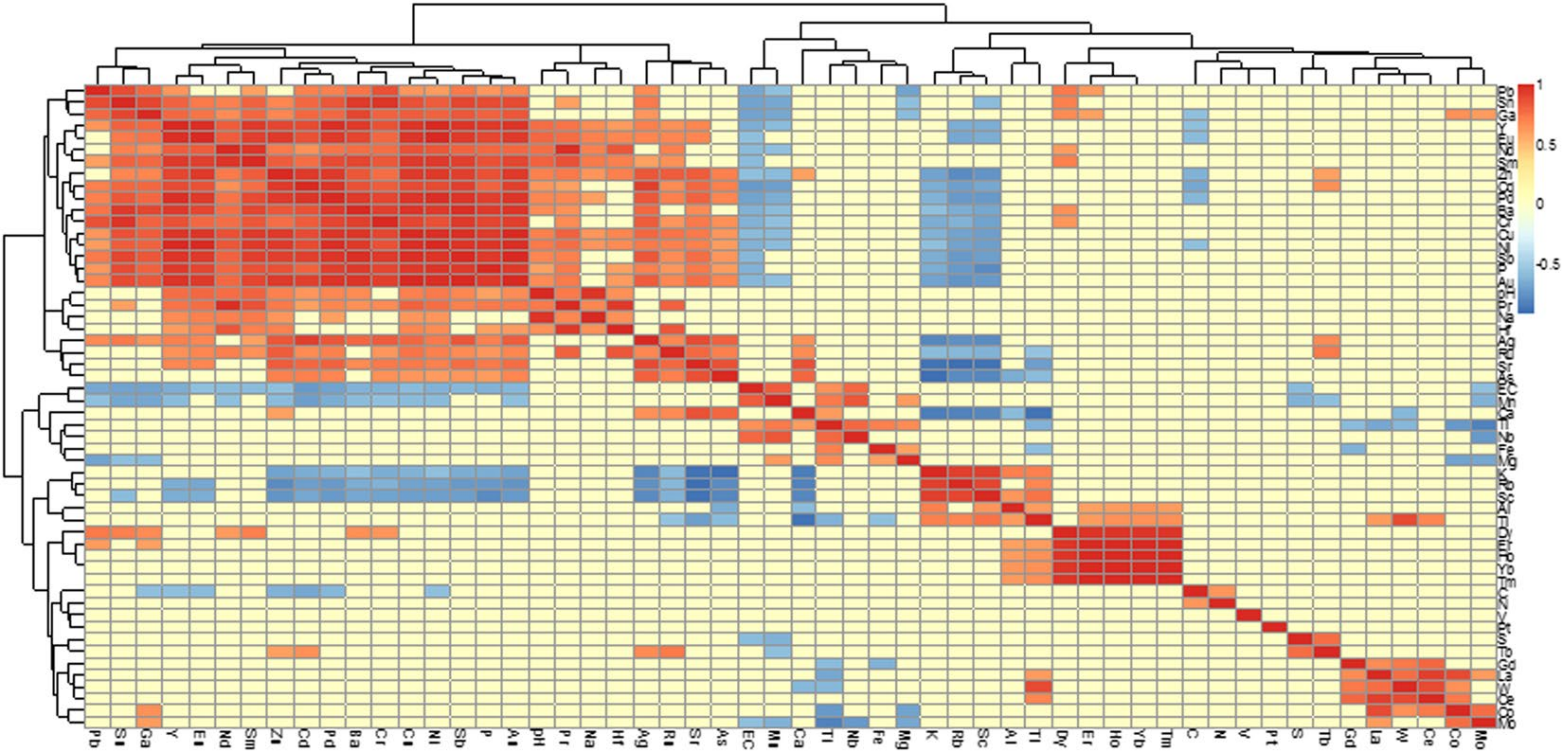
Correlation between elements and environmental factors.

The correlation among the elemental concentrations and environmental parameters was carried out by SPSS. The heatmap (Fig. 5) was produced by R software version 3.2.2 based on Spearman’s correlation matrix. The red and blue colors show the positive and negative correlation, respectively and the deeper color indicates the stronger correlation. The yellow color indicates no significant correlation with *p* value > 0.05. Generally, the elements were clustered into two apparent groups. Five rare earth elements (Pr, Nd, Sm, Eu, and Y) showed strong and positive correlation (r^2^ > 0.5) with eight industrial heavy metals (Pb, Sn, Zn, Cd, Cr, Cu, Ni, Sb). These elements also showed a strong and positive correlation with pH. This could be explained by the fact that the pH plays an important role in the concentration and accumulation of elements in the soil or sludge^35^. Other physicochemical parameters didn’t show any significant correlation with the elements concentrations in the sludge. In addition, rare earth elements, including Dy, Er, Ho, Yb and Tm, also showed strong correlation with each other. The strong correlations among the elements might be due to the similarity in their usage and origin.

#### Geoaccumulation index (*Igeo*)

*Igeo* was used to evaluate the level of soil, sludge and sediment contaminations by comparing with the background concentration of a given element in the studied sample to its concentration in the upper continental crust (UCC) concentration^36, 37^. *Igeo* was calculated via the equation (1)^38, 39^.

1 $$Igeo={\mathrm{log}}_{2}\,(\frac{{\rm{Cn}}}{1.5\,\mathrm{Bn}})$$

Where, Cn is the detected concentration of a given element in the sludge, Bn is the background concentration in the UCC. The factor 1.5 is introduced to minimize the effect of possible variations in the background values which may be attributed to the lithogenic variations in the soil. The enrichment classes (Table S3)^40^ were applied to evaluate the contamination of individual element in each sludge sample.

Generally, the target elements were not accumulated in most of the studied sludge samples (*Igeo* ≤ 1) (Table 3). However, the accumulation of some specific elements was remarkable. For example, Sn showed moderate accumulation in the sludge (1 ≤ *Igeo* ≤ 2), while W and Cd indicated moderate to strong accumulation (1 ≤ *Igeo* ≤ 3) in all the WWTPs. In the samples from W3, there was moderate accumulation of Cr, Cu, and Zn, (0 ≤ *Igeo* ≤ 2). This is noteworthy that some precious metals, including Pd, Au, Ag and Ru, showed strong accumulation (*Igeo* ≤ 4) in the sludge from all WWTPs, indicating their enrichment in the sludge.

**Table 3:** Geoaccumulation index (*Igeo*) values of elements in the sludge samples.

	Bn (mg kg^−1^)*	*Igeo* (W1)	*Igeo* (W2)	*Igeo* (W3)
Al	8.23E + 04	−5.42E-01	−4.40E-01	−5.01E-01
Fe	5.63E + 04	−1.98E-01	−3.26E-01	−2.24E-01
P	2.33E + 04	−3.77E-01	−3.16E-01	1.83E-02
Ca	4.15E + 04	−5.08E-01	−7.24E-01	−4.94E-01
K	2.09E + 04	−9.48E-01	−8.37E-01	−1.07E + 00
Mg	1.05E + 03	5.28E-01	3.88E-01	4.00E-01
Na	2.36E + 04	−1.22E + 00	−1.23E + 00	−1.09E + 00
Mn	9.50E + 02	−3.56E-01	−5.31E-01	−6.73E-01
W	1.90E + 00	**1.64E + 00**	**3.11E + 00**	**2.61E + 00**
Ti	5.70E + 03	−1.04E + 00	−1.36E + 00	−1.19E + 00
Ba	6.28E + 02	−3.95E-01	−3.02E-01	3.39E-03
Sr	3.20E + 02	−4.21E-01	−5.11E-01	−3.59E-01
Zn	6.70E + 01	6.66E-01	3.61E-01	**1.35E + 00**
Cu	2.80E + 01	1.88E-01	5.85E-01	**1.52E + 00**
Sn	2.10E + 00	**1.65E + 00**	**1.84E + 00**	**2.10E + 00**
Ni	4.70E + 01	−6.75E-01	−1.14E-01	9.37E-01
Cr	9.20E + 01	−5.81E-01	−1.62E-01	1.06E + 00
Ga	1.75E + 01	1.07E-01	3.41E-01	4.84E-01
Pb	1.70E + 01	2.25E-01	2.83E-01	3.96E-01
V	9.70E + 01	−9.75E-01	−1.01E + 00	−1.00E + 00
Co	1.73E + 01	−6.77E-01	2.73E-01	1.29E-01
As	4.80E + 00	−2.05E-01	−2.59E-01	−1.41E-01
Rb	8.40E + 01	−5.84E-01	−3.86E-01	−9.96E-01
Nb	1.20E + 01	−3.27E-01	−5.95E-01	−5.85E-01
Mo	1.10E + 00	1.01E + 00	1.40E + 00	1.34E + 00
Cd	9.00E-02	**1.83E + 00**	**2.66E + 00**	**2.64E + 00**
Sb	4.00E-01	2.13E-01	2.58E-01	1.49E + 00
Hf	5.30E + 00	−4.67E-01	−4.63E-01	−2.17E-01
Re	1.98E-01	—	—	—
Tl	9.00E-01	−6.16E-01	−3.97E-01	−5.80E-01
Pd	5.40E-04	**3.47E + 00**	**3.43E + 00**	**4.13E + 00**
Ag	5.30E-02	**1.88E + 00**	**1.43E + 00**	**2.25E + 00**
Au	1.50E-03	**2.73E + 00**	**2.78E + 00**	**3.40E + 00**
Ru	3.40E-04	**2.14E + 00**	**1.98E + 00**	**2.33E + 00**
Ir	2.20E-05	—	—	—
Pt	5.00E-04	**1.54E + 00**	**1.48E + 00**	**1.46E + 00**
Ce	6.30E + 01	−1.05E-01	6.67E-01	3.98E-01
Nd	2.70E + 01	−2.66E-02	8.43E-02	2.47E-01
La	3.10E + 01	−8.69E-02	7.38E-01	5.27E-01
Y	2.10E + 01	−1.13E-01	1.49E-02	6.30E-01
Pr	7.10E + 00	−1.02E-02	9.96E-02	3.18E-01
Sc	1.40E + 01	−1.00E + 00	−7.34E-01	—
Sm	4.70E + 00	1.22E-01	2.00E-01	4.07E-01
Gd	4.00E + 00	2.46E-01	3.85E-01	3.04E-01
Dy	3.90E + 00	3.67E-02	1.07E-01	1.50E-01
Er	2.30E + 00	−3.74E-02	6.71E-02	7.37E-02
Yb	1.96E + 00	5.53E-03	1.18E-01	1.18E-01
Eu	1.00E + 00	9.87E-02	1.83E-01	9.78E-01
Ho	8.30E-01	−4.47E-02	4.80E-02	4.64E-02
Tb	7.00E-01	**1.08E + 00**	**1.06E + 00**	**1.12E + 00**
Tm	3.00E-01	−6.88E-03	1.07E-01	1.08E-01
Lu	5.00E-01	—	—	—

Bn is the concentration of a given element in upper continental crust; *Concentration from^36, 37^.

## Conclusions

In this study, the occurrence of 52 elements was investigated in the sludge samples from three WWTPs in Xiamen city, China over one year. There were 49 elements detected, including major industrial elements, precious metals, and rare earth elements. The concentrations of most elements showed strong spatial variations but little seasonal variations. The spatial variations were mainly due to the difference of the wastewater source as well as treatment processes in WWTPs. Sludge from W1 and W2, which mainly received domestic wastewater, had relatively low elemental concentrations and could be suitable for the agricultural land application after a thorough stabilization and sequestration of potential emerging contaminants and persistent organic pollutants. However, sewage sludge from W3, which received more than 50% of the industrial wastewater, was found with relatively higher elemental concentrations and would pose an environmental risk for the agricultural usage. The high geoaccumulation index in W3 suggested that the elemental recovery might be a better option for the disposal of sewage sludge from W3. The investigation on a broad range of elements in this study provided useful information to assess the environmental risks and to evaluate the potential value of their recoveries, which can be very important for the sludge disposal and management.

## Electronic supplementary material

Supplementary Information
