# Supplementary material for: Assessment of the occurrence, spatiotemporal variations and geoaccumulation of fifty-two inorganic elements in sewage sludge: A sludge management revisit
Source: Sci Rep. 2017 Jul 18;7:5698. doi: 10.1038/s41598-017-05879-9 (PMC5515912; doi:10.1038/s41598-017-05879-9)
Supplement: Supplementary file 1 — Supplementary Information [file 41598_2017_5879_MOESM1_ESM.pdf]

# Supporting information

# Assessment of the occurrence, spatiotemporal variations and geoaccumulation of fifty-two inorganic elements in sewage sludge: A sludge management revisit

Fidèle Suanon[1,2,3], Qian Sun[1,*], Xiaoyong Yang[1], Qiaoqiao Chi[1], Sikandar I. Mulla[1], Daouda Mama[4], Chang-Ping Yu[1,5*]

[1]CAS Key Laboratory of Urban Pollutant Conversion, Institute of Urban Environment, Chinese Academy of Sciences, Xiamen 361021, China
[2]University of Chinese Academy of Sciences, Beijing 100049
[3]Laboratory of Physical Chemistry, University of Abomey-Calavi, BP: 4521 Cotonou, Republic of Benin.
[4]Laboratory of Inorganic Chemistry and Environment, University of Abomey-Calavi, BP: 4521 Cotonou, Republic of Benin.
[5]Graduate Institute of Environmental Engineering, National Taiwan University, Taipei 106, Taiwan

*Corresponding author:

Dr. Qian Sun: Email: qsun@iue.ac.cn

Dr. Chang-Ping Yu: Email: cpyu@iue.ac.cn

**Supplementary Information**

4 pages include 3 tables (Table S1-S3) which directly referred to the text.

**Table S1:** Samples digestion program

| Pressure | Temperature increasing rate/min | Temperature/$^{o}$C | Residence time/min |
|---|---|---|---|
| 1600 (100%) | 10 | 180 | 10 |
| 1600 (100%) | 5 | 180 | 45 |

**Table S2:** Metals recovery (R) and detection limit (DL) of analyzed components

| **Elements** | **DL (µg kg$^{-1}$)** | **Recovery (%)** |
|---|---|---|
| Al | 4.48E-01 | NA |
| Fe | 9.93E-01 | NA |
| P | 1.39E+00 | 96.9 |
| Ca | 4.99E+00 | NA |
| K | 1.32E+01 | NA |
| Mg | 1.06E-01 | NA |
| Na | 8.07E+00 | NA |
| Mn | 1.08E-01 | 93.5 |
| W | 8.57E-02 | 91.5 |
| Ti | 2.1E+00 | 88.3 |
| Ba | 1.82E-01 | 65.8 |
| Sr | 1.73E-02 | 73.5 |
| Zn | 5.43E-01 | 100.9 |
| Cu | 7.49E-01 | 95.6 |
| Sn | 1.06E-01 | 98.8 |
| Ni | 8.70E-01 | 73.2 |
| Cr | 2.87E-01 | 83.37 |
| Ga | 6.7E-02 | 89.5 |
| Pb | 6.88E-02 | 62.3 |
| V | 6.78E-02 | 86.5 |
| Co | 6.47E-01 | 91.2 |

| | | |
|---|---|---|
| As | 5.04E-01 | 92.1 |
| Rb | 3.73E-02 | 88.9 |
| Nb | 7.94E-02 | 68.7 |
| Mo | 5.51E-02 | 98.3 |
| Cd | 5.91E-02 | 76.7 |
| Sb | 4.17E-02 | 96.7 |
| Hf | 5.60E-03 | 58.9 |
| Re | 8.60E-03 | NA |
| Tl | 6.30E-03 | 53.2 |
| Pd | 6.30E-03 | NA |
| Ag | 6.10E-03 | 94.7 |
| Au | 6.30E-03 | NA |
| Ru | 2.60E-03 | NA |
| Ir | 6.30E-03 | NA |
| Pt | 6.20E-03 | NA |
| Ce | 9.10E-03 | 100.2 |
| Nd | 1.80E-03 | 102.5 |
| La | 1.70E-03 | 102.2 |
| Y | 6.60E-03 | 70.1 |
| Sc | 2.24E-02 | 100.7 |
| Pr | 5.70E-03 | 97.7 |
| Sm | 4.80E-03 | 96.3 |
| Gd | 8.40E-03 | 96.3 |
| Dy | 3.90E-03 | 84.6 |
| Er | 2.90E-03 | 76.3 |
| Yb | 5.60E-03 | 89.7 |
| Eu | 1.90E-03 | 90.2 |
| Ho | 1.90E-03 | 79.2 |
| Tb | 4.8E-03 | 82.4 |
| Tm | 3.20E-03 | 89.9 |
| Lu | 4.20E-03 | NA |

**Note:** Element with the mark not available (NA) are the elements which concentrations were not reported in the reference standard material.

**Table S3:** Modified degree of contamination classification and description[40]

| **Value** | **Class** | **Quality** |
|---|---|---|
| $Igeo \leq 0$ | 0 | Uncontaminated |
| $0 \leq Igeo \leq 1$ | 1 | Uncontaminated to moderately contaminated |
| $1 \leq Igeo \leq 2$ | 2 | Moderately contaminated |
| $2 \leq Igeo \leq 3$ | 3 | Moderately to strongly contaminated |
| $3 \leq Igeo \leq 4$ | 4 | Strongly contaminated |
| $4 \leq Igeo \leq 5$ | 5 | Strongly to extremely contaminated |
| $Igeo \geq 5$ | 6 | Extremely contaminated |

**Reference**

40. Müller, G., 1969. Index of geoaccumulation in sediments of the Rhine river. Geojournal 2, 108-118.
